# Artificial intelligence plays an important role in containing public health emergencies

**DOI:** 10.1017/ice.2020.103

**Published:** 2020-04-02

**Authors:** Ningning Tang, Guangyi Huang, Min Li, Fan Xu

**Affiliations:** Department of Ophthalmology, People’s Hospital of Guangxi Zhuang Autonomous Region, Nanning, Guangxi, China

*To the Editor—*Despite measures taken by the Chinese government to contain coronavirus disease 2019 (COVID-19), its transmission has yet to be halted. Instead, the SARS-Cov-2 virus has demonstrated international seeding leading to a global outbreak.^1^ Although many technical gaps and uncertainties remain in the epidemic management techniques, artificial intelligence (AI) offers the potential for concerned parties to fill these gaps using big data.

Currently, thanks to the support of the Ministry of Industry and Information Technology of China,^2^ research institutions and enterprises across China are actively optimizing AI algorithms and computing power for epidemic control. For example, an automated whole-genome sequencing and analysis platform has been established to promote rapid and accurate virus detection.^3^ Additionally, protein screening, as well as new drugs and vaccine development, have been accelerated thanks to the use of AI.^4^ By applying image-processing algorithms, chest computed tomographic images can be intelligently diagnosed and quantitatively evaluated, and the severity of various types of pneumonia (ranging from local lesions and diffuse lesions to whole-lung involvement) are automatically graded.^5^ Case assessments for COVID-19 using AI systems show acceptable accuracy with high efficiency.^5^ Phonetic interface systems and speech-recognizing robots are frequently employed in healthcare settings to provide intelligent guidance and triage services.^6^ In some communities and public places, these systems can replace healthcare workers in performing some routine tasks, such as temperature measurement, that can be done automatically and accurately through a combination of deep learning, image recognition technology, and infrared imaging sensors.^7^ Smart-dial service can be used employed to conduct phonetic verification with preset conversation content (eg, health status, travel, and contact history), so that residents’ health conditions and population mobility can be efficiently investigated.^8^ Facial recognition technology is used to track behavior via real-time processing of video data of close contacts of confirmed and/or suspected cases.^9^ All in all, the application of AI technology has opened up opportunities for the integration and utilization of big data across the whole of Chinese society, providing a wealth of information for hospital management, community supervision, and government policy formulation.

Efforts made by a single country remain insufficient. Although the world is currently facing the COVID-19 epidemic, other events, such as a natural disaster followed by a pandemic, might occur in the future as well. In such an event, worldwide data sharing and technology communication will be required to both create and launch a global, public health emergency response plan and to effectively contain the public health emergency. We hope that an open AI-based platform, with epidemiological database integration and comprehensive coverage worldwide, will be established to maximize the benefits of AI and big data. In this way, the nations of the world can cooperate more closely to effectively respond to the common crises that face all of mankind.
